# Suture-based rib approximation for the repair of minimally invasive surgery-related lung hernia: a case series

**DOI:** 10.1093/jscr/rjaf518

**Published:** 2025-07-15

**Authors:** Yuki Takahashi, Masahiro Miyajima, Ryunosuke Maki, Yoshiki Chiba, Takeshi Ohyu, Kazuki Sato, Kazuya Honda, Atsushi Watanabe

**Affiliations:** Department of Thoracic Surgery, Sapporo Medical University, School of Medicine and Hospital, South 1, West 16, Chuo-ku, Sapporo, Hokkaido 060-8556, Japan; Department of Thoracic Surgery, Sapporo Medical University, School of Medicine and Hospital, South 1, West 16, Chuo-ku, Sapporo, Hokkaido 060-8556, Japan; Department of Thoracic Surgery, Sapporo Medical University, School of Medicine and Hospital, South 1, West 16, Chuo-ku, Sapporo, Hokkaido 060-8556, Japan; Department of Thoracic Surgery, Sapporo Medical University, School of Medicine and Hospital, South 1, West 16, Chuo-ku, Sapporo, Hokkaido 060-8556, Japan; Department of Thoracic Surgery, Sapporo Medical University, School of Medicine and Hospital, South 1, West 16, Chuo-ku, Sapporo, Hokkaido 060-8556, Japan; Department of Thoracic Surgery, Sapporo Medical University, School of Medicine and Hospital, South 1, West 16, Chuo-ku, Sapporo, Hokkaido 060-8556, Japan; Department of Thoracic Surgery, Sapporo Medical University, School of Medicine and Hospital, South 1, West 16, Chuo-ku, Sapporo, Hokkaido 060-8556, Japan; Department of Thoracic Surgery, Sapporo Medical University, School of Medicine and Hospital, South 1, West 16, Chuo-ku, Sapporo, Hokkaido 060-8556, Japan

**Keywords:** lung hernia, minimally invasive surgery, minimally invasive cardiac surgery, video-assisted thoracoscopic surgery

## Abstract

Lung hernia is a rare complication of minimally invasive surgery (MIS). Suture-based rib approximation to restore costal margin continuity during MIS-related lung hernia repair has not been reported. We present three cases of repair using suture-based rib approximation for MIS-related lung hernias. Between 2016 and 2025, our department performed four MIS-related lung hernia repairs. Three followed minimally invasive cardiac surgery (MICS), and one followed video-assisted thoracoscopic surgery (VATS). We excluded the MICS case requiring third-rib resection during MICS and Gore-Tex patch for repair. All MICS-related hernias occurred at the right fourth intercostal space; the VATS-related hernia occurred at the left fourth intercostal space. In all three cases, hernial defects were repaired with sutures placed across the intercostal space between the upper and lower ribs. No perioperative mortality, morbidity, or recurrence occurred. Suture-based rib approximation demonstrated favorable outcomes. Further case accumulation is necessary to identify risk factors and establish optimal techniques.

## Introduction

Minimally invasive surgery (MIS) has become increasingly common in thoracic surgery. Among these approaches, minimally invasive cardiac surgery (MICS) for cardiac procedures and video-assisted thoracoscopic surgery (VATS) for pulmonary procedures have gained widespread adoption worldwide [1, 2]. Although rare, lung hernia has been reported as a complication of MIS [3, 4]. To date, no standardized method has been established for the repair of lung hernias.

In cases of lung hernia following MIS, repair using autologous tissues or synthetic materials such as Vicryl, polytetrafluoroethylene, Dacron, Marlex, or Gore-Tex patches has been reported. However, to date, no studies have documented the use of rib approximation with sutures alone for the repair of postoperative lung hernias [3, 4].

In this study, we aimed to report successful outcomes achieved using rib approximation with sutures alone for the repair of lung hernias following MIS, highlighting the restoration of costal margin continuity.

## Case presentations

### Case 1

**Figure 1:**
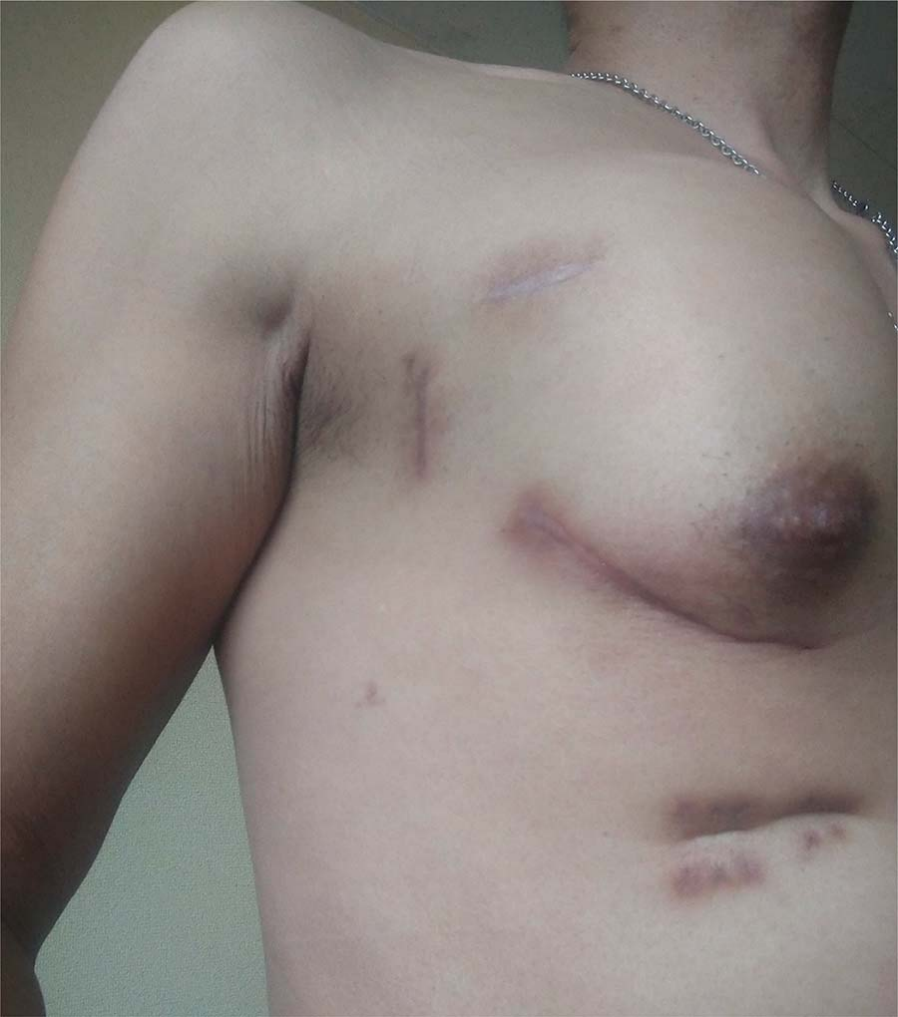
Swelling in the right anterior chest due to a lung hernia.

**Figure 2:**
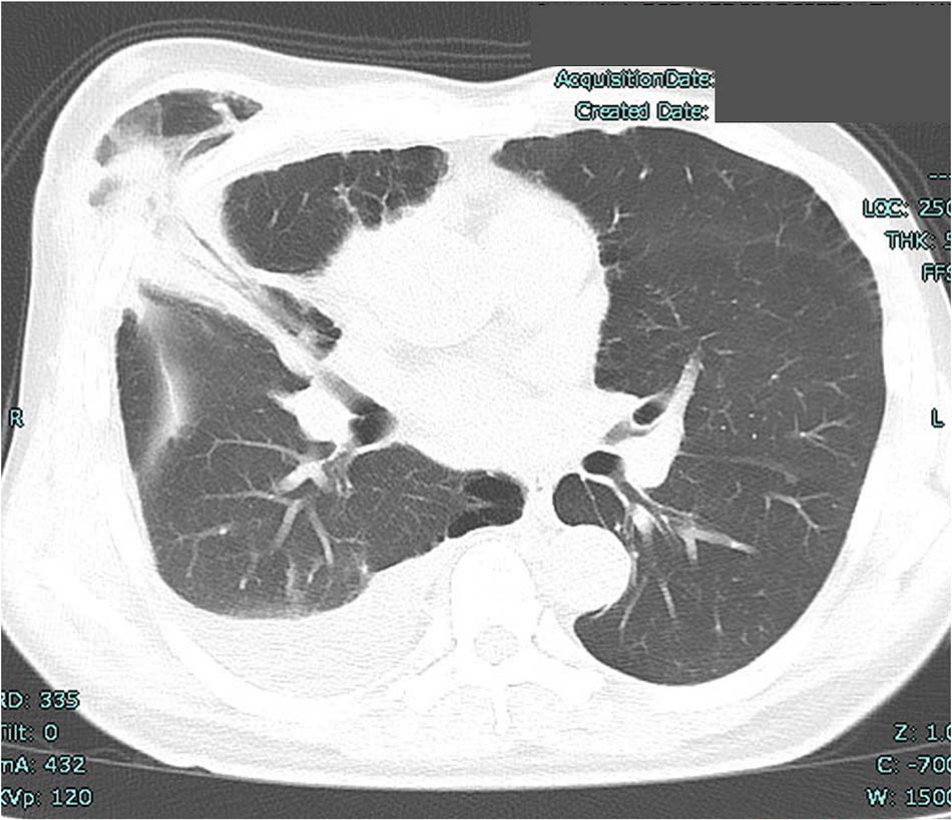
Lung herniation of the right middle lobe at the fourth intercostal space.

A 53-year-old man underwent mitral valve repair (MVR) via MICS to treat mitral regurgitation. One month postoperatively, the patient presented with swelling of the right anterior chest (Fig. 1). High-resolution computed tomography (HRCT) revealed lung herniation at the fourth intercostal space (ICS), corresponding to the incision site of the previous MICS procedure on the right chest wall (Fig. 2).

**Figure 3:**
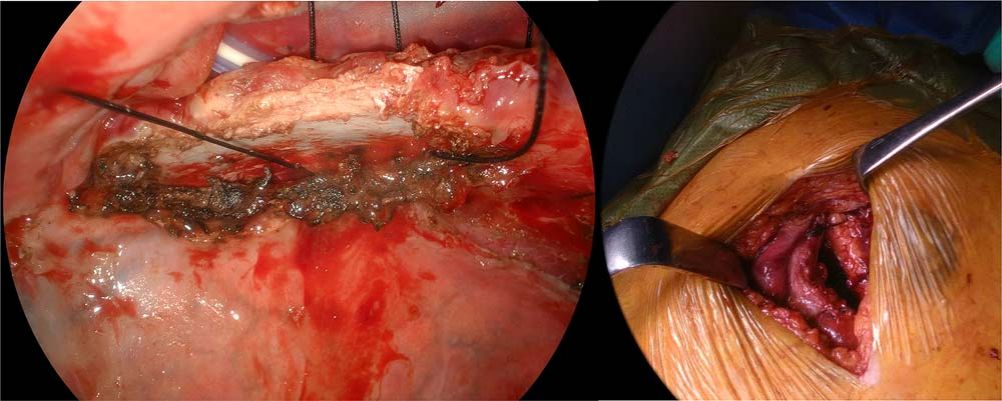
Intraoperative view of rib approximation using nonabsorbable sutures.

The patient underwent anterior thoracotomy at the site of the hernia, followed by scar resection of the previous incision. Additionally, a 12-mm port was placed at the sixth ICS to facilitate the observation of the thoracic cavity. The hernial defect was repaired exclusively with four stitches of size one nonabsorbable silk suture placed across the ICS between the fourth and fifth ribs, forming the hernial orifice (Fig. 3). Prior to suturing, the adhesion between the right upper lobe and fourth rib was meticulously dissected as extensively as possible. Resection of the herniated lung tissue was not required.

**Figure 4:**
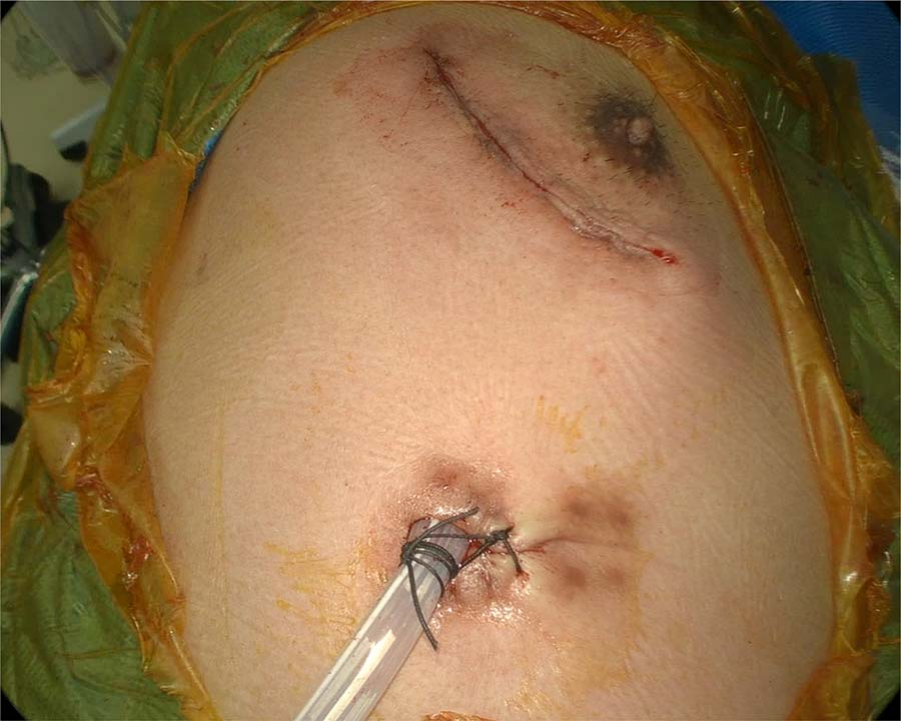
Chest drain placed through the port incision following the repair.

The wound was closed in separate layers, including the closure of the pectoralis muscles, deep dermal layer, and subcuticular layer, using running absorbable sutures (Fig. 4). A chest drain was then placed. No recurrence of the lung hernia was observed during the 6-month follow-up period.

### Case 2

A 55-year-old woman underwent MVR via MICS for mitral regurgitation. One year postoperatively, the patient presented with a worsening bulge upon straining. HRCT revealed lung herniation at the fourth ICS, corresponding to the incision site of the previous MICS procedure on the right chest wall.

The patient underwent an anterior thoracotomy at the site of the hernia, followed by scar resection of the previous incision. In addition, a 5-mm port was placed at the sixth ICS to facilitate the observation of the thoracic cavity. The hernial defect was closed exclusively with four stitches of 2-0 nonabsorbable polyester sutures (Ti-Cron™) placed across the ICS between the fourth and fifth ribs, forming the hernial orifice. Prior to suturing, the adhesion between the right upper lobe and the fourth rib was meticulously dissected without requiring resection of the herniated lung tissue.

The wound was closed in layers using absorbable running sutures. No chest drains were placed. During the 9-year follow-up, the patient showed no recurrence of the lung hernia, demonstrating long-term procedural success.

### Case 3

A 77-year-old man underwent a left lingular segmentectomy via VATS to treat squamous cell carcinoma. Five months after surgery, the patient presented with a progressively enlarging bulge upon straining. HRCT revealed lung herniation at the fourth ICS, corresponding to the previous VATS incision site on the left chest wall.

The patient underwent an anterior thoracotomy at the site of the hernia, followed by scar resection of the previous incision. Additionally, a 12-mm port was placed at the sixth and seventh ICS to assist thoracic cavity visualization. A pleural fistula in the left upper lobe was identified and repaired. The hernial defect was repaired exclusively with four stitches of size one nonabsorbable polyester suture (Ti-Cron™) placed across the ICS between the fourth and fifth ribs, forming the hernial orifice.

The wound was closed with absorbable sutures. A chest drain was then placed. During the 7-year follow-up, no recurrence of the lung hernia was observed, confirming the long-term efficacy of the repair.

## Discussion

Lung hernias following MIS are rare complications. To date, 35 cases of lung hernia have been reported after MIS, 31 of which occurred after MICS (Table 1) [3–10]. This finding indicates that lung hernias are more frequently reported following MICS than following VATS.

**Table 1:** Previous reports of MIS-related lung hernia

First author	Country	Year	Number of patients	Types of MIS	Sex	Age (years)	Side and ICS of hernia	Length of MIS incision (mm)	Methods of hernia repair
Ng PC [5]	USA	2000	3	MICS	Unknown	Unknown	Lt. 4th ICS	80–100	2 underwent patch repair 1 treatment unknown
Temes RT [6]	USA	2001	1	VATS	Male	68	Lt. ICS unknown	30	No surgical repair
Athanassiadi K [3]	Germany	2008	16	MICS	12 males, 4 females	23–77	8 Rt., 6 Lt. 2 bilateral ICS unknown	50–80	10 underwent patch repair 6 surgical repair (details unknown)
Kumar N [7]	Netherlands	2013	1	MICS	Female	62	Lt. 6th or 7th ICS	30	Surgical repair (details unknown)
Koichi Y [8]	Japan	2019	1	MICS	Female	51	Rt. 4^th^ ICS	70	No surgical repair
Batihan G [4]	Turkey	2020	3	VATS	2 males, 1 female	58–65	1 Rt., 1 Lt. 1 side unknown 4th or 7th ICS	30–60	All patients underwent patch repair
Nanjo K [9]	Japan	2023	1	MICS	Male	51	Rt. 2nd ICS	Unknown	Underwent patch repair
Vinck EE [10]	Colombia	2024	9	MICS	4 males, 5 females	51–77	5 Rt. 2nd ICS 4 Rt. 4th ICS	Unknown	Underwent patch repair
Takahashi Y (our cases)	Japan		3	2 MICS 1 VATS	2 males, 1 female	53–77	2 Rt. 4th ICS 1 Lt. 4th ICS	60–80	All patients underwent rib approximation with sutures

MIS, minimally invasive surgery; MICS, minimally invasive cardiac surgery; VATS, video-assisted thoracoscopic surgery; ICS, intercostal space; Lt., left side; Rt., right side

Lung hernias following MIS have been reported at various ICS, including the 2nd, 4th, 6th, and 7th ICS. However, in all three cases in our study, the hernias involved were located in the 4th ICS. Furthermore, the reported incision lengths for MIS-related lung hernias range from 30 to 100 mm. In our cases, the incision length was 60–80 mm (Table 2).

**Table 2:** Patient characteristics and treatment for MIS-related lung hernia repair in our cases

No.	Types of MIS	Sex	Age (year)	Side and ICS of hernia	Length of MIS incision (mm)	Methods of hernia repair
1	MICS	Male	53	Rt. 4th ICS	80	Rib approximation with sutures
2	MICS	Female	55	Rt. 4th ICS	80	Rib approximation with sutures
3	VATS	Male	77	Lt. 4th ICS	60	Rib approximation with sutures

MIS, minimally invasive surgery; MICS, minimally invasive cardiac surgery; VATS, video-assisted thoracoscopic surgery; ICS, intercostal space; Lt., left side; Rt., right side

In terms of repair methods, surgical treatment was performed in 32 cases, whereas conservative treatment was employed in three cases. Among the 32 surgically treated cases, 25 used synthetic materials, such as poly-L-lactide (u-HA/PLLA) mesh plates, monofilament polypropylene mesh, Vicryl, polytetrafluoroethylene, or Gore-Tex patches, for hernia repair. Athanassiadi et al. stated that rib approximation and restoration of costal margin continuity are feasible only in cases of early detected traumatic hernias. However, in our study’s cases of MIS-related lung hernias, satisfactory outcomes were achieved using rib approximation with sutures alone. This approach eliminates the need for costly synthetic patches, potentially reducing medical costs.

Preventing pulmonary hernias following MICS during closure is considered critical [3]. Among the preventive strategies, the suture-based rib approximation technique reported in this study may serve as an effective method for achieving this goal.

## Conclusion

Suture-only rib approximation repair demonstrated favorable outcomes for MIS-related lung hernias in patients who did not require rib resection. This method proved to be a cost-effective and efficient alternative to synthetic patches. However, further case accumulation is essential to investigate the risk factors for the development of MIS-related lung hernias and establish optimal surgical techniques for synthetic patch-based repairs.

## Data Availability

The datasets used or analyzed during the current study are available from the corresponding author upon reasonable request.
